# Investigating Male Presence at Antenatal and Choice of Place for Child Delivery in Ghana

**DOI:** 10.3389/fpubh.2019.00300

**Published:** 2019-10-22

**Authors:** Phidelia Theresa Doegah

**Affiliations:** Institute of Health Research, University of Health and Allied Sciences, Ho, Ghana

**Keywords:** maternal health, male presence, antenatal, place of delivery, Ghana

## Abstract

Male involvement in maternal health was introduced to improve and sustain maternal and child health in Ghana. The study utilized the 2014 Ghana Demographic and Health Survey data to investigate the relationship between male presence at antenatal and choice of place of childbirth among 1,167 males, 15–59 years. Descriptive and analytical statistical techniques were applied to the data. The binary logistic regression shows no association between male presence at antenatal and place of delivery (OR = 1.197; 95% CI = 0.808–1.773). However, age (OR = 2.647; 95% CI = 1.221–5.736, OR = 3.046; 95% CI = 1.345–6.896, OR = 3.513; 95% CI = 1.478–8.345), level of education (OR = 4.478; 95% CI = 1.412–14.1990, religion (OR = 0.473; 95% CI = 0.237–0.946), ethnicity (OR = 0.400; 95% CI = 0.182–0.877, OR = 0.425; 95% CI 0.194–0.935), marital status (OR = 5.682; 95% CI = 2.093–15.421, OR = 5.669; 95% CI = 1.448–22.198), place of residence (OR = 7.272; 95% CI = 4.231–12.499), and region of residence (OR = 11.515; 95% CI = 2.785–47.618) of males were found associated with health facility based delivery. Regarding policy to promote institutional delivery among women, these socio-demographic factors identified should be considered.

## Introduction

In 2015, about 303,000 women were reported to have died from pregnancy and childbirth around the world. The majority of these deaths were noted to occur in developing countries (1). Global efforts to reduce maternal mortality rate (MMR) by <70% in 2030 are embodied in the sustainable development goal (SDG) number 3. Ghana, following global efforts, also had in place several strategies in a bid to save the lives of mothers and children. Strategies include, among others, the launch of free antenatal and delivery care for pregnant women, Safe-Motherhood initiative, Prevention of Maternal Mortality Program (PMMP), Maternal and Neonatal Health Program, and Making Pregnancy Safer Initiative. However, the MMR was reducing at a slow pace and thus Ghana missed out on the 2015 millennium development goal (MDG) to reduce MMR by 75% (2).

Ghana's Ministry of Health (MoH), together with the United Nations Fund for Population Activities (UNFPA), realized the dominance of males in the Ghanaian context, and introduced the concept of male involvement in order to promote family planning, gender equality, and maternal and child health (3). In the African setting, the man is the head of the house and is largely responsible for all decision making, or every decision is approved by him, as most husbands are financially more empowered in comparison to their wives. As such, there is the need to involve them in maternal issues if women are to patronize the services. For instance, in studies on male involvement in family planning, scholars found among Nigerian males in Ile-Ife Osun State that their poor involvement in family planning (FP) decision resulted in low use of FP services [(4), p. 49]. Additionally, a study in Tanzania found that women in male headed households usually deliver out of health facilities [(5), p. 867–868]. There is a consensus that when males know and can identify symptoms related to pregnancy complications then they are able to act upon the knowledge and seek skilled care for the woman [(6), p. 33].

Antenatal care (ANC) and use of skilled birth attendants are essential to reduce maternal mortality (MM) as complications can be detected during these visits by the skilled attendants. However, in Ghana, recognizing the critical signs and knowing what to do are usually part of the ANC session for the pregnant women, and thus the need for males to be present for spouse's ANC arises. Positive male involvement in maternal health then refers to the mental and physical participation of males in maternal and prenatal health and family planning in such a way as to increase maternal and fetal survival rates (7).

Pregnancy or child bearing from anecdotal sources has been regarded solely as a woman's duty, as such studies have focused largely on background characteristics of women such as age, educational level [(8), p. 6]; place of residence [(8), p. 7]; and occupational status [(5), p. 865]. It is generally assumed that the use of maternal healthcare services such as the choice of place to deliver is dependent on the woman. Due to this assumption, there has been less research on male involvement in maternal health.

As observed, fewer studies to date have investigated whether male presence at ANC culminated into choice of health facility for child delivery using the Ghanaian population. Results of this relationship would be vital for policy to promote the utilization of maternal healthcare services to increase maternal and fetal survival. Using the Ghana Demographic and Health Survey (GDHS) 2014 data, this study sought to determine whether male presence at ANC determines choice of place of child delivery.

Nevertheless, individual male socio-demographic characteristics could also predict the place of children's birth and these were included as a control for anticipated male presence effects. A study has found that a man's background characteristics also play a role in a woman's choice of place [(9), p. 103]. For instance, a husband's level of education was found to be associated with wives' use of maternal health care services in Nigeria and Ghana respectively [(10, 11), p. 3]; also, rural residences of males were found to be associated with lower participation in maternal healthcare [(12), p. 153]. However, Danforth et al. [(13), p. 701]; Carter and Speizer [(12), p. 153] observed no association between a woman's use of maternal services and her husband's level of education amongst a sample of women in Tanzania. Also associated with maternal healthcare use is the region of the residence of males (10). This research seeks to address the following research questions: does a male presence at ANC result in the choice of health facility for child delivery? What male socio-demographic factors are associated with the choice of health facility for child delivery controlling for male presence at ANC?

## Materials and Methods

Data for this study are from the 2014 Ghana Demographic and Health Survey (GDHS). The GDHS (14) is a household-based survey with a probability sample of 12,831 households selected nationwide and stratified by region and residence. It employed a two-stage sampling design where the first stage involved selecting sample points or clusters from an updated master sampling frame constructed from the 2010 Ghana Population and Housing Census. A total of 427 clusters were selected from the master sampling frame. The clusters were selected using systematic sampling with probability proportional to size. The second stage of selection involved the systematic sampling of 30 of the households listed in each cluster.

Three questionnaires were used in all: the Household, the Women's, and the Men's Questionnaires. The Men's Questionnaire was administered to all men aged 15–59 living in half of the selected households in the GDHS sample. A sample of 1,139 (1,167 un-weighted sample) males was extracted for this study through two stages. At the first instance, respondents with missing cases on variable mv250 (place of birth of most recent child) were dropped, a total of 3,178 observations. Secondly, respondents with missing data for mv249 (respondent present during check-ups for most recent child) were also dropped (total of 43 observations dropped), and thus arrived at the final sample size. To ensure representativeness and correct non-responses, the data is weighted taking into consideration the complex survey design, using the “svyset” command in Stata. The survey set is adjusting for complex survey, cluster, strata, and weight variable. The variables required for the “svyset” is the wtvar (weight variable), Primary Sampling Unit (PSU), and Strata. The “svyset” command used in Stata for this study is svyset [pw = wtvar], psu (mv021) strata (mv023).

The independent variable for the study is male presence at antenatal *(if man was present during wife's antenatal for most recent child [Yes/No])* and the outcome variable is the choice of delivery place of child *(“Was [NAME] born in a hospital or health facility?” the responses were hospital/health facility or other)*. For the outcome variable, the “health facility” response was re-coded “1” and “Other” re-coded “0.” The “Other” option entails any place other than a health facility.

Ghana Statistical Service (GSS) the institution responsible for the GDHS data collection obtained ethical clearance from the relevant Institutional Review Boards (IRBs) and consent from the eligible respondents.

### Data Analysis

Variables were explored with the use of descriptive statistics (frequencies and percentages). Bivariate analysis (cross tabulations) was carried out to examine the variations in the study sample regarding place of delivery of child and explanatory variables. The Chi-Square test statistic was used to assess the significance between background variables and place of delivery. Significance was set at 0.05. To examine the influence of male presence on place of delivery a binary logistic regression was fitted. The model results were interpreted using the odds ratio (OR) and the _hatsq (hat square) value was used to determine a well-specified model. All analyses were conducted using Stata version 12.0.

## Results

In Table 1, generally more than half (56%) of males indicated absenteeism during spouses' ANC attendance. Results show that a higher proportion (40%) of men belonged to the 25–34 age group whilst the age group 15–24 had the least proportion. More than a half of the sample had attained a Senior Secondary School level with the least proportion with a higher education. More than three-fifth (66%) of the men are Christians, followed by males who are Muslim, whilst a lesser proportion belonged to the “Other” religious group. A higher proportion of the sample is made up of Akan, Mole-Dagbani and “Other” accordingly. Males' in-union constituted the most, whilst the least proportion is formerly married. A greater number of the men reside in rural areas. In terms of region of residence, a higher proportion of males in the study sample dwell in the Greater Accra region followed by Ashanti region whilst the least stays in the Upper West region. Majority (80%) of the sample indicated their children were delivered in a health facility.

**Table 1:** Percentage distribution of respondents background characteristics.

**Variables**	**Frequency**	**Percentage (%)**
	1,139	100.00
**PRESENT AT ANC**		
Yes	494	43.4
No	645	56.6
**AGE**		
15–24	56	5
25–34	457	40.1
35–44	453	39.8
45–59	172	15.1
**EDUCATIONAL LEVEL**		
No education	215	18.9
Primary	153	13.4
Secondary	657	57.6
Higher	114	10
**RELIGION**		
Christians	758	66.6
Islam	261	22.9
Traditional/spiritualist	57	5
No religion	63	5.5
**ETHNICITY**		
Akan	508	44.6
Ga/Dangme	65	5.7
Ewe	143	12.5
Mole-Dagbani	225	19.7
Other	199	17.5
**MARITAL STATUS**		
Not in union	33	2.9
In union	1084	95.2
Formerly in union	22	1.9
**PLACE OF RESIDENCE**		
Urban	543	47.7
Rural	596	52.3
**REGION OF RESIDENCE**		
Western	119	10.5
Central	120	10.5
Greater accra	203	17.8
Volta	88	7.7
Eastern	93	8.1
Ashanti	196	17.2
Brong-Ahafo	114	10
Northern	132	11.6
Upper east	46	4
Upper west	29	2.6
**PLACE OF BIRTH**		
Health facility	915	80.3
Other	224	19.7

*Source, Computed from GDHS 2014 male data file*.

Results in Table 2 show the higher the level of education among males, the more children delivered in a health facility. For instance, men with a higher level of education had the majority (95%) of their children delivered in a health facility, compared to children of males with no formal education (60%). Christian (84.93%), Islamic (74.23%), and males of “Other” (72.53%) religious belonging have more children delivered in a health facility. Males of Akan (87.56%), Ga/Dangme (84.13%), and Ewe (83.54%) ethnic origins have more than three quarters of children delivered in a health facility. In terms of marital status, males not in union (single) or formerly married have the highest number of children (92.17 and 91.95%, respectively) delivered in a health facility. Urban dwelling males have the most children (95.12%) delivered in a health facility. Majority of males who reside in the Upper East (94.44%), Greater Accra (93.86%), and Ashanti (91.48%) regions have most of the children born in a health facility whilst males resident in the Northern region have the least (49.75%) of children delivered in a health facility.

**Table 2:** Percentage distribution of respondents by place of delivery, presence at ANC, and background characteristics.

**Background characteristics**	**Place of delivery**		**Number of men**	
	**Health facility**	**Others**		
	**(%)**	**(%)**	**Freq**	**%**
**PRESENT AT ANC**				
Yes	82.8	17.2	493	100
No	78.4	21.6	645	100
χ^2^-value = 3.3850; *P* = 0.1593				
**AGE**				
15–24	69.7	30.3	56	100
25–34	80.8	19.2	457	100
35–44	80.9	19.1	453	100
45–59	81.0	19.0	172	100
χ^2^-value = 4.3368; *P* = 0.3666				
**EDUCATIONAL LEVEL**				
No education	60.1	39.9	215	100
Primary	74.9	25.1	153	100
Secondary	85.6	14.4	656	100
Higher	95.3	04.7	114	100
χ^2^-value = 88.5058; *P* < 0.001				
**RELIGION**				
Christians	84.9	15.1	758	100
Islam	74.2	25.8	260	100
Traditional/Spiritualist	55.1	44.9	57	100
Other	72.5	27.5	63	100
χ^2^ value = 42.5568; *P* = 0.0001				
**ETHNICITY**				
Akan	87.6	12.4	508	100
Ga/Dangme	84.1	15.9	65	100
Ewe	83.5	16.5	143	100
Mole-Dagbani	72.7	27.3	224	100
Other	66.8	33.2	199	100
χ^2^ value = 50.8452; *P* = 0.0001				
**MARITAL STATUS**				
Not in union	92.2	07.8	33	100
In union	79.7	20.3	1084	100
Formerly in union	91.9	08.1	22	100
χ^2^ value = 5.1669; *P* = 0.0390				
**PLACE OF RESIDENCE**				
Urban	95.1	04.9	543	100
Rural	66.8	33.2	596	100
χ^2^ value = 147.5551; *P* < 0.001				
**REGION OF RESIDENCE**				
Western	77.7	22.3	119	100
Central	77.8	22.2	120	100
Greater Accra	93.9	06.1	203	100
Volta	79.3	20.7	88	100
Eastern	74.6	25.4	93	100
Ashanti	91.5	08.5	196	100
Brong Ahafo	78.4	21.6	114	100
Northern	49.8	50.2	132	100
Upper East	94.4	05.6	46	100
Upper West	77.3	22.7	29	100
χ^2^-value = 129.3370; *P* < 0.001				
Total No.	915	224	1139	
%	80.3	19.7		100

*Computed from GDHS 2014 male data file*.

To ascertain how well male presence determines the choice for a health facility as a place for child delivery, a single predictor binary logistic regression was fitted. Results shown in Table 3 indicate males not present at ANC are less likely (0.085) compared to those present at ANC to have children delivered in a health facility. A second model fitted introduced socio-demographic factors of males as control variables with the results shown in Table 4. From the Table, males within the age groups 25–34 (2.647), 35–44 (3.046), or 45–59 (3.513) are more likely to have their children delivered in a health facility compared to children of males within the age bracket 15–24 years. Males with a higher level of education (4.478) are more likely than males with no education to have their children delivered in a health facility.

**Table 3:** Binary logistic regression model showing the association between male presence at ANC and health facility delivery.

**Variable**	**Odds ratio**	**S.E**	**95%**	**CI**
**MALE PRESENT AT ANC**				
Present (RC)	1.000			
Not present	0.085^*^	0.144	−0.198	0.368

* *<0.05. Computed from the GDHS 2014 male data file*.

**Table 4:** Binary logistic regression analysis of association between ANC presence, socio-demographic characteristics, and delivery in health facility.

**Variable**	**Odds ratio**	**S.E**	**95%**	**CI**
**MALE PRESENT AT ANC**				
Present (RC)	1.000			
Not present	1.197	0.239	0.808	1.773
**AGE**				
15–24 (RC)	1.000			
25–34	2.647*	1.041	1.221	5.736
35–44	3.046*	1.266	1.345	6.896
45–59	3.513*	1.546	1.478	8.345
**EDUCATIONAL LEVEL**				
No education (RC)	1.000			
Primary	1.179	0.378	0.628	2.214
Secondary	1.379	0.449	0.727	2.618
Higher	4.478*	2.628	1.412	14.199
**RELIGION**				
Christian (RC)	1.000			
Islam	0.947	0.344	0.463	1.934
Traditional/spiritualist	0.473*	0.167	0.237	0.946
No religion	0.647	0.195	0.358	1.169
**ETHNICITY**				
Akan (RC)	1.000			
Ga/Dangme	0.505	0.266	0.179	1.424
Ewe	0.653	0.224	0.333	1.281
Mole-Dagbani	0.400*	0.160	0.182	0.877
Other	0.425*	0.170	0.194	0.935
**MARITAL STATUS**				
Never in union	5.682*	2.885	2.093	15.421
In union (RC)	1.000			
Formerly in union	5.669*	3.935	1.448	22.198
**PLACE OF RESIDENCE**				
Urban	7.272*	2.003	4.231	12.499
Rural (RC)	1.000			
**REGION OF RESIDENCE**				
Western	0.617	0.351	0.201	1.888
Central	0.599	0.372	0.176	2.035
Greater Accra (RC)	1.000			
Volta	0.806	0.473	0.254	2.558
Eastern	0.537	0.271	0.199	1.447
Ashanti	1.710	1.092	0.487	6.003
Brong-Ahafo	0.716	0.405	0.236	2.175
Northern	0.460	0.282	0.138	1.536
Upper East	11.515*	8.312	2.785	47.618
Upper West	2.125	1.376	0.595	7.591
* <0.05	_hatsq = 0.383			

*Source, Computed from GDHS 2014 data file*.

Males who belong to the Traditional/Spiritualist religion are less likely (0.473) than their Christian counterparts to have children delivered in a health facility. Mole-Dagbani or “Other” males compared to Akan males are less likely (0.400 and 0.425, respectively) to have children delivered in a health facility.

The never married males are more likely (5.682) than married males to have children delivered in a health institution. Males residing in urban areas compared to those in the rural areas are 7.272 more likely to have children delivered in a health facility. Compared to males in the Greater Accra region, males resident in the Upper East region are 11.515 more likely to have children delivered in a health facility.

## Discussion

This study contributes to literature on male participation in maternal and child health in the Ghanaian context using the 2014 Ghana Demographic and Health Survey. Generally, result indicates male presence at ANC did not determine the choice of place of child delivery, contrary to the expectation that when males accompany spouses to ANC the health facility will be the place of choice for delivery. However, findings among Kenyans and Indians indicate a higher facility delivery among women whose male partners were present during ANC attendance [(15), p. 375; (16), p. 143]. Probably because it is not an integral part of the Ghanaian setting for husbands to be involved in their wife's ANC or their maternal health issues. In the Ghanaian society, men are congratulated and praised upon the conception of their spouses but rarely expected to be involved in issues concerning the pregnancy. Issues concerning pregnancies are considered a woman's business and not of concern to the man. This may explain why a man's presence during ANC is not a determinant of choice of place of child delivery.

Increasing age is found to predict the choice of health facility as a place of delivery. This is contrary to findings from Myanmar [(17), p. 6] and Indonesia [(18), p. 1,206]. Age is correlated to maturity as such within the Ghanaian setting, with increasing age, males may have observed outcomes from facility and out of facility delivery. This may have influenced their inclination toward a facility based delivery.

Level of education specifically higher education was found significant in predicting a health facility as a choice of place for delivery. Formal education is expected to make individuals much more knowledgeable on the merits of utilizing the maternal healthcare services which entails health facility delivery. The current study's finding about the positive effect of education on place of delivery corroborates an existing study [(10, 11), p. 3]. However, Danforth et al. [(13), p. 701] identified no relationship between the educational level of males and the choice of place of delivery.

Traditional/spiritualist males are found to be less likely to have their children delivered in a health facility. This is contrary to a finding by IIiyasu et al. [(19), p. 27] in Nigeria where religion was not found to predict delivery in a health facility. People who profess this religion are adherents to traditional ways of life and are opposed to almost everything modern and what it brings. They are more in support of the use of herbs and traditional birth attendants, among others. They are thus more likely then to prefer delivery in their own home or the home of others such as that of a medicine man or woman.

In terms of ethnicity, males belonging to Mole-Dagbani or “Other” ethnic groups were found to be less likely to have their children delivered in a health institution. Similarly, a study by IIiyasu et al. [(19), p. 27] also found ethnic variation among ethnic groups in Nigeria. The various ethnic groups have different social and cultural practices in relation to childbirth which are practiced by the individual group members. This most likely may affect the choice of place of delivery.

Males who had never married were found to have a higher likelihood of having children delivered in a health facility. Study by IIiyasu et al. [(19), p. 27] found the marital status of male partners insignificant in the choice of a health facility for child delivery in Nigeria. Probably, as parents who are not married, they are independent in decision making regarding the pregnancy and ultimately the choice of delivery place. Urban residence among males is also associated with facility delivery. This is consistent with a study by Kurniati et al. [(18), p. 1,206] who also found urban residence associated with facility delivery in Indonesia. This may be explained by the fact that urban areas are clustered with both public and private health facilities in addition to ease of transportation. In addition, health facility delivery maybe an urban lifestyle compared to a home based delivery.

The region in which respondents' dwell in affects the place of child delivery as men in the Upper East region have more children delivered in a health facility. This may be explained by the presence of the Demographic and Health Surveillance System (DHSS) in the region as a result individuals are prompted to seek health care. Additionally, the region because of the DHSS has been the first to receive several interventions such as Community and Health Planning Services (CHPS) which has brought health services closer to the populace.

In terms of limitations, findings and conclusions are based on self-reports of respondents other than objective measures, as responses may have been influenced by social desirability. Also, causality cannot be established due to the cross-sectional nature of the data.

In conclusion, the study has shown that although male presence has no association with choice of place of child delivery, male background factors determine the choice of place for child delivery. In the face of efforts to improve maternal and child health, males need to be largely involved in governmental efforts.

### Policy Implication

The identification of male background characteristics to determine the choice of place of child delivery indicates males should be involved in maternal health issues. The identified background factors should be considered in all maternal and child health related policy interventions. For instance, given the fact that education is vital to the choice of place of delivery, there is the need to promote and intensify health education in men with no formal education or less than a higher level of education. Secondly, considering the variation in religion, marital status, ethnicity, place, and region of residence regarding choice of place of delivery, specific tailored interventions must be formulated to address these variations. Interventions could consider how to integrate these factors into policies or as a leveraging tool to increase institutional delivery.

## Data Availability Statement

Data used for the study is available upon request from Measure DHS.

## Ethics Statement

This study made use of the Ghana Demographic and Health Survey (GDHS 2014) and no mention was made of the IRB that granted ethical approval.

## Author Contributions

PD conceived the study, run the analysis, wrote, and finalized the manuscript for submission.

### Conflict of Interest

The author declares that the research was conducted in the absence of any commercial or financial relationships that could be construed as a potential conflict of interest.
